# Cocoa Seeds as a Phytobezoar Causing Intestinal Obstruction in a Ghanaian Child: A Case Report

**DOI:** 10.7759/cureus.76562

**Published:** 2024-12-29

**Authors:** Lawrence Nsohlebna Nsoh, Akosua K Asiedu-Asante, Hussein A Yakubu, Kwaku Nyame

**Affiliations:** 1 Emergency Medicine Department, Komfo Anokye Teaching Hospital, Kumasi, GHA

**Keywords:** bezoars, case report, children, cocoa seed, ghana, intestinal obstruction, komfo anokye teaching hospital, phytobezoar

## Abstract

Phytobezoars are indigestible organic matter that forms organized masses in the gastrointestinal tract. Seeds reported causing bezoars include sunflower seeds, watermelon seeds, and wild banana seeds. Cocoa seeds causing bezoar have not been reported. The seeds tend to have an outer shell that cannot be digested by human digestive enzymes. Cocoa seed has a shell made of pectic polysaccharide, hemicellulose, and cellulose, which cannot be digested by human digestive enzymes. Accumulation of these seeds and fibers swallowed or chewed in some parts of the small intestine, the colon, and rectum, with fluid absorption in these parts of the tract, forms hard bezoars in the tract and can sometimes lead to intestinal obstruction. Reported cases of seed phytobezoar follow the geographical distribution of the seed or fruit, the dietary habits of the people, and the seasonal pattern of maturity of the seeds and fruit.

Patients present with non-specific abdominal symptoms. Intestinal obstruction is a rare complication, and if present, the patient will present with associated constipation, vomiting, and abdominal distension. Known predisposing factors to bezoar formation from phytobezoars include previous gastric surgery, neuropsychiatric disorder, and endocrinopathies, even though the majority will have no risk factors. Diagnosing involves a good history and examination. Anoscopy or colonoscopy is used in selected cases to identify gastrointestinal masses as an underlying cause of bezoar formation. CT scan is the gold standard, and other imaging, like abdominal X-ray and ultrasound, may be useful in diagnosing and locating the bezoar and complications in a limited resource environment. Management includes manual evacuation under anesthesia if the bezoar is in the rectum. Targeted deimpaction with enzymes may be considered in some cases. Bezoars higher up in the tract or with complications of ischemia or perforation will require surgery or endoscopy.

We present a rare case of cocoa seeds bezoar causing intestinal obstruction in a six-year-old child from a rural town in the Asante Region of Ghana, presenting to Komfo Anokye Teaching Hospital in Ghana, West Africa.

## Introduction

Phytobezoars are indigestible organic matter that forms organized masses in the gastrointestinal tract [1]. They consist of indigestible plant fibers such as seeds, roots, leaves, orange pits, or pulpy fruits. Seeds such as sunflower seeds, watermelon seeds, wild banana seeds, cocoa seeds, or any other fruit seed can form a bezoar when ingested. Seed phytobezoars, which are a subset of phytobezoars, can sometimes cause intestinal obstruction [1].

Cocoa is a common crop in southern Ghana. On occasion, ripe cocoa seeds are consumed as snacks in local communities. Similar to other grains and beans, cocoa seeds are not digested if swallowed whole. Due to their tiny size, they may pass through the pylorus and ileocecal valves, eventually accumulating in the small bowel, colon, and rectum to form hard beads [2]. The cocoa bean consists of an outer shell or husk, which is a by-product of the processing of the bean. Chocolate, which is the main product of cocoa, does not contain more than 5% of the shell as a standard required for production. The shell has very useful nutrients, including protein, fatty acids, vitamins, flavanols, minerals, methylxanthines, and both digestible and indigestible fiber. The indigestible fiber constitutes 28.34 g to 50.42 g per 100 g of cocoa beans. The indigestible fiber is made of pectic polysaccharide, hemicellulose, and cellulose. When the seed or bean is swallowed whole, human digestive enzymes are unable to break down the beans due to the high content of the indigestible fiber of the shell. This is further aggravated by fluid reabsorption, which occurs in the gastrointestinal tract, leading to hard bean formation and accumulation, together forming a bezoar and causing intestinal obstruction, especially in children [1-6].

We present a rare case of cocoa seeds causing intestinal obstruction in a six-year-old male child without any underlying disease from a remote village in the Asante Region of Ghana, presenting to Komfo Anokye Teaching Hospital in Ghana, West Africa.

## Case presentation

**Figure 1:**
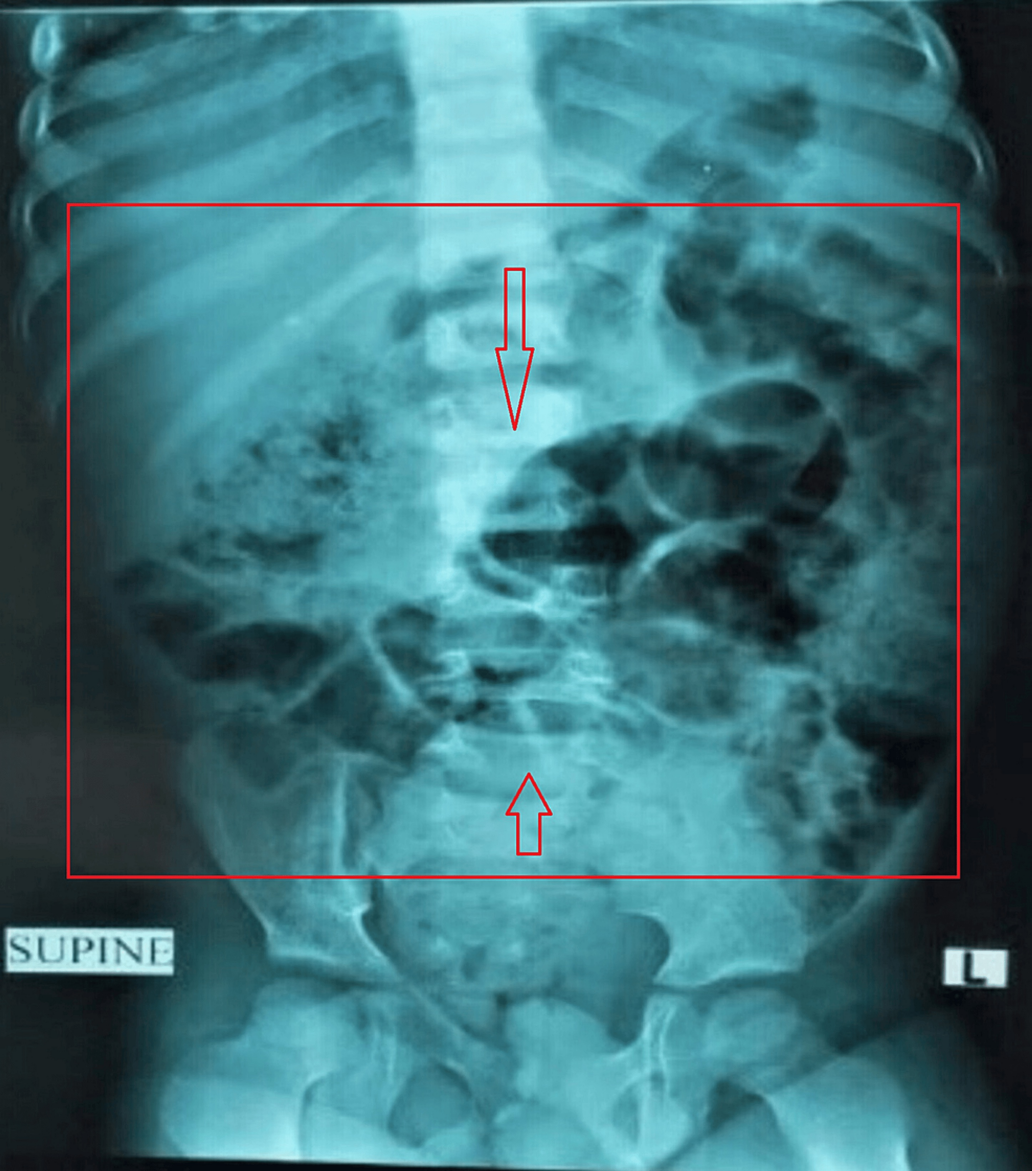
Supine abdominal X-ray before evacuation

A six-year-old male with no significant history presented to the Komfo Anokye Teaching Hospital Emergency Department with a day history of abdominal pain and constipation, associated with non-bilious, non-projectile vomiting containing food previously eaten. His vital signs were within the normal range for his age. An abdominal examination revealed a distended abdomen with mild generalized tenderness but no signs of peritonitis. The rectal examination with attempted disimpaction was significant for hard, irregular, fecaloid-like masses, some of which turned out to be cocoa seeds along with stool. Investigations done included a supine abdominal X-ray, which showed a ground glass appearance with a dilated loop of bowel (see Figure 1).

**Figure 2:**
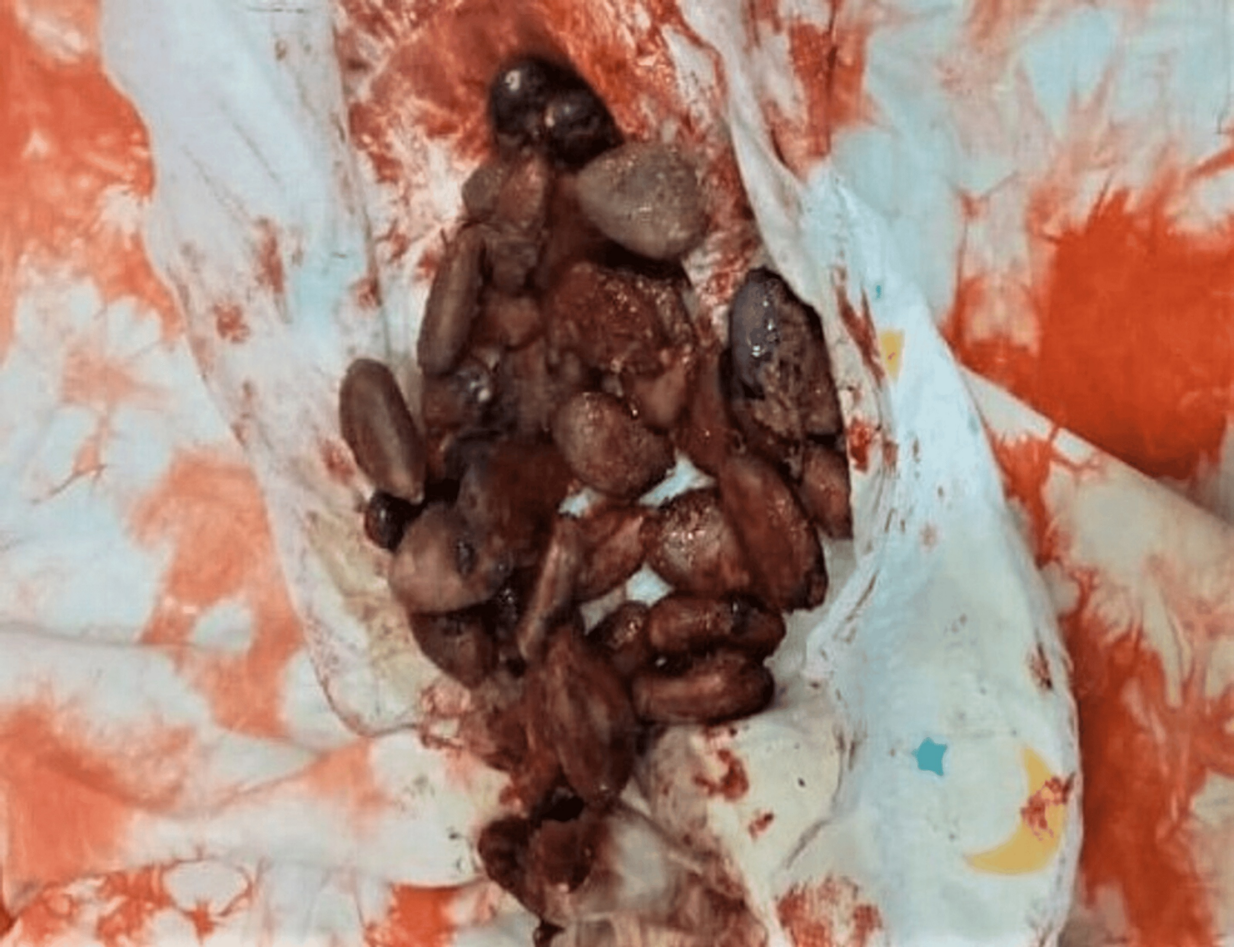
Cocoa seeds manually evacuated in the operating theater

An abdominal ultrasound scan further revealed a dilated rectum with compacted fecal matter. All other lab work including complete blood count, liver function test, and renal function test were within normal limits. A clinical diagnosis of acute intestinal obstruction from cocoa seed phytobezoars was made. The pediatric surgery team was consulted, and the final decision was made to do manual evacuation and colonic lavage under general anesthesia. Anal dilatation, manual evacuation, and colonic irrigation were performed under general anesthesia on the same day. About 30 g of cocoa seeds of various sizes were evacuated (see Figure 2).

The patient was admitted to the hospital and observed for 48 hours after the manual evacuation. Follow-up erect and supine X-rays of the abdomen were normal with no clinical symptoms and signs of intestinal obstruction; thus he was discharged home. The patient was re-examined a week after discharge and had abdominal X-rays performed, which were unremarkable.

## Discussion

Seed phytobezoars, which are a subgroup of phytobezoars, can, on rare occasions, cause intestinal obstruction [3,4,7,8]. It is caused by a buildup of indigestible vegetable or fruit seeds in the digestive lumen. Because of their small size, grains and seeds normally pass through the pylorus and ileocecal valve and gradually accumulate in the intestine. When the fecal mass reaches the rectum, it is further dried and hardened into a bezoar, which frequently manifests as fecal impaction. This pathologic mechanism of seed phytobezoars is compatible with our example in which the intestinal blockage in the rectum was caused by eating cocoa seeds as a snack. Most seed phytobezoars occur in patients without predisposing factors [1,3-8]. However, some are associated with risk factors such as previous gastric surgery, neuropsychiatric disorders, and endocrinopathies (diabetes, hypothyroidism), accounting for approximately 12% of all reported cases [1]. The reported case had no known risk factor, which is consistent with the majority of cases reported with seed phytobezoar.

Reported cases of seed phytobezoar follow the geographical distribution of the seed or fruit, the dietary habits of the people, and the seasonal pattern of maturity of the seeds and fruit. Documented cases of seed phytobezoar causing intestinal obstruction include watermelon and prickly pear, sunflower, pumpkin, wild banana, and mango seeds. The cases are commonly found in various geographical locations in different seasons [1]. Cocoa seeds are seasonal fruit common among the farming communities in the southern part of Ghana. The presented case was a child who lived in a cocoa-farming rural area where cocoa seeds are sometimes consumed as a snack, especially by children. This occurred during the prime of the cocoa season in Ghana. 

Children and adults both had 84% and 70% more seed phytobezoars in the rectum, respectively [1]. Children with seed bezoar typically appear with constipation as their primary symptom (69% of children and 56% of adults), which is followed by non-specific stomach or rectal discomfort (19% of children and 17% of adults) [1]. Intestinal obstruction is uncommon (17% of all documented instances) and mostly affects individuals with terminal ileum seed bezoars [3]. Other rare clinical symptoms include abdominal distention, nausea, and vomiting. The patient whom we presented had clinical symptoms such as constipation, abdominal pain, and rectal pain, which is consistent with other seed bezoar presentations.

In diagnosing seed phytobezoars, a detailed history and thorough physical examination are usually indicated [1-4,7-10]. Anoscopy can help with some cases of digital per rectum examination, which is essential to the diagnostic process. To rule out malignant disease, which may not be necessary for children, a thorough colonoscopy may be advised in adult patients. Other tests, like plain abdominal radiography and abdominal ultrasounds, may detect a solid mass in the stools or may typically provide normal results. The gold standard for diagnosis is a computed tomography scan, which provides details on the kind, location, and severity of the blockage as well as any possible ischemia of the intestinal wall [1,2]. A CT scan was not performed on this patient because there were no clinical signs of peritonitis and a desire to minimize radiation exposure. The diagnosis of seed phytobezoar causing the intestinal obstruction was based on the detailed history suggestive of seed phytobezoar, followed by a digital rectal examination that revealed cocoa seeds in the rectum. Abdominal X-rays supported the diagnosis of intestinal obstruction without air under the diaphragm, which could have suggested perforation, and ultrasound was done, which helped locate the seed bezoar collection.

Colonic bezoars are treated differently depending on the colon's location, the kind, and the size of the seed phytobezoar [1,2]. Rectal seed phytobezoar is most effectively managed by manual evacuation while under general anesthesia. If this method is unsuccessful, and there is colonic damage or intestinal perforation evident at presentation, surgery is the best option. In this instance, we successfully did manual seed removal from the colon while under general anesthesia.

In cases of phytobezoar with underlying diseases like previous gastrointestinal surgery and endocrinopathies, there is a likelihood of recurrence, and the patient should be advised on a dietary modification that includes a low-fiber diet and prophylactic medication to enhance gastrointestinal emptying [9,10]. Our patient did not have an underlying disease. The cocoa seeds evacuated were unchanged, which is indicative that the child swallowed the seed whole and was indigestible, leading to the bezoar formation and intestinal obstruction. Communities should be made aware that the cocoa seed, if swallowed whole, is indigestible and could lead to bezoar formation.

## Conclusions

Cocoa seeds, when swallowed whole, can lead to seed phytobezoar formation due to the indigestible shell of the seed, and on rare occasions may subsequently lead to intestinal obstruction. Diagnosing phytobezoars requires a detailed history of diet habits as well as seasonal and geographical seeds consumed. Ultrasound may be useful in resource-limited settings to identify the bezoar, and a digital rectal examination can identify rectal bezoars. Rectal phytobezoars without bowel perforation or ischemia can successfully be managed with manual extraction performed under general anesthesia.

## References

[1] Manatakis DK, Acheimastos V, Antonopoulou MI, Balalis D, Korkolis DP (2019). Gastrointestinal seed bezoars: a systematic review of case reports and case series. Cureus.

[2] Senol M, Ozdemir ZÜ, Sahiner IT, Ozdemir H (2013). Intestinal obstruction due to colonic Lithobezoar: a case report and a review of the literature. Case Rep Pediatr.

[3] Sealey AJ, Lau NS (2024). Peanut phytobezoar: an unusual cause for small bowel obstruction. J Surg Case Rep.

[4] Natarajan DK, Homthavong P, Trehan I (2019). Small bowel obstruction secondary to wild banana seed ingestion. Am J Trop Med Hyg.

[5] Edo GI, Samuel PO, Oloni GO (2023). Review on the biological and bioactive components of cocoa (Theobroma cacao). Insight on food, health and nutrition. Nat Resour Hum Health.

[6] Rojo-Poveda O, Barbosa-Pereira L, Zeppa G, Stévigny C (2020). Cocoa bean shell - a by-product with nutritional properties and biofunctional potential. Nutrients.

[7] Law GW, Lin D, Thomas R (2015). Colonic phytobezoar as a rare cause of large bowel obstruction. BMJ Case Rep.

[8] Hargrave RL, Hargrave R (1936). Acute intestinal obstruction by the Persimmon phytobezoar: report of two cases. Ann Surg.

[9] Olumide F, Adedeji A, Adesola AO (1976). Intestinal obstruction in Nigerian children. J Pediatr Surg.

[10] Teng H, Nawawi O, Ng K, Yik Y (2005). Phytobezoar: an unusual cause of intestinal obstruction. Biomed Imaging Interv J.

